# Middle-range theory for nursing for care in the context of cardiovascular risk

**DOI:** 10.1590/0034-7167-2024-0190

**Published:** 2024-09-20

**Authors:** Nuno Damácio de Carvalho Félix, Alba Lúcia Bottura Leite de Barros, Maria Miriam Lima da Nóbrega

**Affiliations:** I Universidade Federal do Recôncavo da Bahia. Santo Antônio de Jesus, Bahia, Brazil; II Universidade Federal de São Paulo. São Paulo, São Paulo, Brazil; III Universidade Federal da Paraíba. João Pessoa, Paraíba, Brazil

**Keywords:** Nursing Theories, Theoretical Models, Heart Disease Risk Factors, Concept Formation, Standardized Nursing Terminology, Teorías de Enfermería, Modelos Teóricos, Factores de Riesgo de Enfermedad Cardiaca, Formación de Concepto, Terminología Normalizada de Enfermería

## Abstract

**Objectives:**

to develop a middle-range nursing theory for care in the context of cardiovascular risk.

**Methods:**

a theoretical development study, through induction through research and ICNP^®^ practice standards, carried out in six stages: concept analysis; ICNP^®^ terminological subset structuring; theory contextualization and purpose; proposition construction; modeling; and assumption construction.

**Results:**

the Theory of Care in the Context of Cardiovascular Risk has a middle-range scope, describing care, prescribing actions to promote health and reduce cardiovascular risk. Thirteen propositions were constructed in three categories (nursing metaparadigm, central and factorial concepts), two models and 16 assumptions.

**Final Considerations:**

the theory contributes to the construction of knowledge arising from the nursing process for care in the context of cardiovascular risk, generating propositions, assumptions and modeling, which will enable theoretical testing.

## RESUMEN

**Objetivos:**

desarrollar una teoría de rango medio de enfermería para el cuidado en el contexto de riesgo cardiovascular.

**Métodos:**

estudio de desarrollo teórico, mediante inducción a través de la investigación y estándares de práctica de la CIPE^®^, realizado en seis etapas: análisis de concepto; estructurar el subconjunto terminológico de la CIPE^®^; contextualización y finalidad de la teoría; construcción de proposiciones; modelado; y construcción de supuestos.

**Resultados:**

la Teoría de la Atención en el Contexto del Riesgo Cardiovascular tiene un alcance medio, describiendo la atención, prescribiendo acciones para promover la salud y reducir el riesgo cardiovascular. Se construyeron 13 proposiciones en tres categorías (conceptos de metaparadigma enfermero, central y factorial), dos modelos y 16 supuestos.

**Consideraciones Finales:**

la teoría contribuye a la construcción de conocimientos derivados del proceso de enfermería para el cuidado en el contexto de riesgo cardiovascular, generando proposiciones, supuestos y modelaciones, que posibilitarán la contrastación teórica.

## INTRODUCTION

The world population, including Brazil, underwent processes of socioeconomic and epidemiological transformation in the last century. The insertion of countless technologies resulting from modernization favored the improvement of people’s quality of life, due to greater comfort in daily life, but it also affected habits related to cardiovascular risk, which crucially influence the health-disease process^(1)^ and the knowledge developed about care for these people. Along with these processes, professional disciplines, including nursing, have sought to develop from the perspective of keeping up with advances as well as improving healthcare and communication to reduce the impacts of these advances, formulating new policies and theories in the field of health.

Clinical nursing practice demands the development of one’s own knowledge, especially regarding care for people at cardiovascular risk, considering that they imply vulnerability, which contributes to the emergence of diseases and complications, which are often irreversible. The fundamental role of the multidisciplinary team, especially nurses, stands out in order to identify, intervene, prevent and produce knowledge about cardiovascular risk factors^(2)^.

There is a need for investigations to describe care for people at cardiovascular risk based on evidence and based on nursing theories directed and induced from clinical practice, with concepts defined and linked to the nursing process and practice standards, using nursing classification systems. In this context, middle-range theories (MRTs) are part of the nursing knowledge base to guide practice and research, and their continued use offers the potential to test and evolve nursing knowledge^(3)^.

The development of a MRT involves critical analysis of the body of knowledge produced, which needs to be organized and described to support nursing care for a specific population; in the case of this study, people with cardiovascular risk. Nursing care based on its own theory contributes to constructing the science and nursing discipline, as the theoretical bases of a science come from a dynamic and creative process, developed to explain and describe related to practice-related items^(4)^. Therefore, considering the magnitude of cardiovascular diseases, especially in the Brazilian context, it is important that nurses access and use theoretical-conceptual support from the discipline that allows rational and objective planning and assessment of interventions to control risk factors^(5)^, impacting the population’s cardiovascular health.

MRTs involve relatively concrete and specific phenomena, stating what they are, why they occur and how they occur, providing support for understanding behavior, situations, events and the connection between nursing phenomena (nursing diagnoses, outcomes and interventions)^(6)^. The process of developing an MRT is presented by inducing standards of nursing practice and research structured in the International Classification for Nursing Practice (ICNP^®^) terminological subset.

Specifically regarding the development of MRT through practice standards, its function is to describe, explain, or predict phenomena in an explicit and testable way, unlike grand theories^(7)^. However, there is a lack of studies with this design and existing evidence, which is still fragmented, has flaws in the explanatory or descriptive connection structure regarding cardiovascular risk, its factors and necessary care.

MRT development using a nursing classification system expresses the discipline, with the intersubjective understanding of the meaning of the concepts included in professional language, and the organization of the concepts that constitute it in a common universe of nursing perception and communication^(8)^. Thus, the present study can contribute to constructing the body of nursing knowledge, with the components described and a concrete theoretical-conceptual framework, feasible and testable in care aimed at people at cardiovascular risk.

## OBJECTIVES

To develop a nursing MRT for care in the context of cardiovascular risk.

## METHODS

### Ethical aspects

As this is a theoretical study, submission to a Research Ethics Committee was not required, but the principles of research with human beings were considered, as they induced the MRT of practice and research.

### Study design

This is a theoretical development study, oriented towards the elaboration of an MRT, using as a strategy induction through research and practice standards arising from the ICNP^®^ terminological subset.

### Methodological procedures

Theory induction occurred through six stages: 1) concept analysis; 2) ICNP^®^ terminological subset structuring; 3) theory contextualization and purpose; 4) proposition construction; 5) theory modeling; and 6) theoretical assumption construction. The innovation in the method developed in this study stands out, with a limited literature on the MRT induction process based on ICNP^®^.

Initially, the stages of concept analysis^(9-10)^ and ICNP^®^ terminological subset structuring^(11-13)^ provided support for the initial induction of an MRT. The contextualization and description of MRT purpose explained the development, relating it to the specific sociopolitical and clinical-epidemiological context descriptively of nursing care linked to non-relational propositions and assumptions aligned with the main phenomenon in the context of cardiovascular risk, metabolic syndrome, justifying the use of analysis of this concept as the first empirical basis of the theory.

In relation to the construction of MRT propositions, there is a relationship between two or more concepts, and they are used to connect concepts in the creation of theories before making explanations or predictions^(7)^. These may be non-relational, corresponding to the definitions of concepts, declaring some type of relationship between two or more concepts and may have an association or causal relationship^(14)^. Considering the strategy of this stage, the definition of the main concepts (cardiovascular risk and metabolic syndrome) and factorial concepts conditioning and associated with cardiovascular risk was presented, correlated to the nursing metaparadigm concepts with philosophical influence from the discipline’s frameworks^(15-16)^.

The theory modeling stage involves schematic representation of the relationships between MRT concepts, presenting aspects of reality about MRT phenomena through various means, such as geometric shapes and diagrams. In this study, two theoretical models were carried out that represent the real world view of MRT phenomena using language or symbols and directional arrows^(7)^.

In modeling, the intellectual and reflective production on MRT phenomena arising from previous stages was considered, with concepts presented in an image structure coherent with the definitions, propositions and assumptions constructed, using the NBR 8403 Standard^(17)^ for scaling lines for use in technical drawings and similar documents, with graphic design support with nursing training and expertise in the field of cardiovascular nursing.

Assumptions involve factual assertions known through experience or may reflect value positions that imply what is right, what is good or should be^(14)^. From this perspective, MRT assumptions were constructed through the body of knowledge analyzed and structured by the previous stages^(9-13)^, which support MRT testing.

## RESULTS

### Theory contextualization

The Theory of Care in the Context of Cardiovascular Risk (*Teoria do Cuidado no Contexto de Risco Cardiovascular*), called by the authors as TEORISC, has the scope of nursing MRT, of the descriptive type, and was developed through several studies linked together and articulated for identification, conceptual and content analysis, structuring and clinical application of an ICNP^®^ terminological subset, in which all components were induced from research and practice standards from the nursing process.

TEORISC is inserted as a theoretical foundation to guide nursing and healthcare at a time when there are high rates of morbidity and mortality due to chronic non-communicable diseases, especially cardiovascular diseases and diabetes *mellitus*, situations that can be preventable by changing habits and reducing risk factors. The theory contributes to advancing knowledge about care directed to the context of cardiovascular risk by presenting relevant aspects about nurses’ actions in the process of reducing risk factors in a sociopolitical moment that demands increasing the visibility of the discipline by consolidating the body of knowledge and generating health indicators related to nursing practice.

The production of knowledge arising from a central phenomenon - metabolic syndrome - in a health context - cardiovascular risk - is combined with organization and description of relevant factors and phenomena directly or indirectly. The nursing actions that are part of TEORISC are linked to a classification system that includes nursing diagnoses, outcomes and interventions that are in a process of constant improvement at national and international levels: ICNP^®^.

TEORISC components are presented, defined and represented in models that facilitate understanding of the knowledge produced. Concepts and their relationships, propositions and assumptions are discussed regarding their relationship with reality and existing knowledge, their impact on healthcare services and indication for use in practice and research, in order to promote testing and generation of evidence regarding the impact of TEORISC on cardiovascular health in various populations in Brazil and around the world.

### Theory purposes

TEORISC describes care in the context of cardiovascular risk, the relationship of conditioning and associated cardiovascular risk factors that precede and increase a person’s vulnerability. There is also a prescription for actions to promote health and reduce cardiovascular risk aligned with individual factors and phenomena to reduce morbidity and mortality from chronic non-communicable diseases in the medium and long term.

### Theory propositions and modeling

TEORISC is made up of 13 propositions, with their respective definitions, organized into three categories involving nursing metaparadigm, central and factorial concepts (conditioning and associated) for care in the context of cardiovascular risk, as described in Chart 1.

**Chart 1:** Theory of Care in the Context of Cardiovascular Risk propositions, João Pessoa, Paraíba, Brazil, 2022

TEORISC propositions	
Concepts	Definitions
Nursing metaparadigm	
Person	Holistic, open, integrated, adaptable and complex human being in a context of cardiovascular risk in their environment/health context and who presents human and social needs (physical, biochemical, intellectual, psychosocial and cultural).
Health	Mind, body and soul unity, with independent functionality, adapted and with its best health condition in the face of cardiovascular, intrinsic and extrinsic stressors and other risks in the health context.
Environment	Internal and external, concrete and abstract elements of an open system that affect the person, their organism and the care and health context.
Nursing	Science, art and practical discipline that involves care using scientific foundations in the theoretical-practical framework construction. Identifies contexts, factors and phenomena relevant to cardiovascular health through the use of clinical reasoning in the decision-making process.
Central concepts	
Cardiovascular risk	Health and care context that allows identifying groups with risk factors for cardiovascular diseases, modifiable (cardiometabolic, behavioral, psychosocial and cultural, occupational, emotional and therapeutic) and non-modifiable (biological) that act as early and interrelated markers of multiple and heterogenic etiology that predispose the person and their community to vulnerability.
Metabolic syndrome	Aggregation of significant cardiovascular risk markers, of multifactorial etiology, related to asymptomatic inflammation that predisposes the person to vulnerability. It involves the identification of at least three diagnostic criteria, such as increased abdominal circumference, increased fasting vascular blood glucose, blood pressure, triglycerides, and/or reduced high-density cholesterol, according to the parameter adopted and the demand for a multidisciplinary approach, including nursing.
Factorial concepts	
Biological	Non-modifiable factors related to a person’s intrinsic life, inserted by time of existence, genetics and/or heredity. They involve phenomena such as sex/gender, age, ethnicity, family history.
Cardiometabolic	Factors related to cardiovascular function, hormonal and nutritional processes, modifiable depending on the specific characteristics of risk phenomena, with an impact on a person’s health. They involve phenomena such as nutrition, body weight, estrogen and progesterone concentration, menopause, sleep and rest.
Behavioral	Modifiable factors related to the individual and/or collective way of proceeding when faced with social and/or affectionate stimuli that compromise cardiovascular health and/or care. They involve phenomena such as eating habits, physical activity/exercise, tobacco/alcohol use, low adherence, therapeutic abandonment, health management and self-care, sexual process and sexuality.
Psychosocial and cultural	Factors involving psychological, social and cultural aspects, modifiable depending on interest, disposition, collective context, self-perception or personal belief, which together compromise cardiovascular health and/or care. They involve phenomena such as knowledge, communication, acceptance and adaptation to health conditions, interpersonal and family relationships, self-image, self-esteem, socioeconomic and cultural conditions, stress, anxiety, spiritual and religious beliefs.
Occupational	Modifiable factors related to the work process, its organization and problems arising from a person’s occupational exhaustion, which compromise health and/or cardiovascular care.
Emotional	Factors related to specific diseases and/or impact on a person’s body, modifiable depending on the availability of treatment for a cure, which compromise cardiovascular health and/or care.
Therapeutic	Factors related to processes, technologies and procedures to treat illnesses that compromise cardiovascular health and/or care, modifiable through intervention in the therapeutic process or cessation of failures and/or errors in management. They involve phenomena such as the use of specific medications, polypharmacy, hormone deprivation or replacement or special treatments.

In TEORISC, a person is related to the human being with cardiovascular risk factors that condition the development of illness (cardiometabolic, behavioral and affective) and associated with each other or with other factors (biological, psychosocial and cultural, occupational and therapeutic), demanding care to prevent chronic non-communicable diseases, promote health and maintain a healthy life, identified by evidence of the central phenomenon. Health should be the purpose of care in the context of cardiovascular risk to avoid, control or eliminate impairment of the central phenomenon and/or promote health and prevent cardiovascular diseases.

In the environment, nursing is inserted with a view to reducing/eliminating cardiovascular risk and its central phenomenon, using a standardized language, prescribing nursing actions to reduce cardiovascular risk, eliminate the central phenomenon and promote health and prevent cardiovascular diseases and other chronic non-communicable diseases.

**Figure 1:**
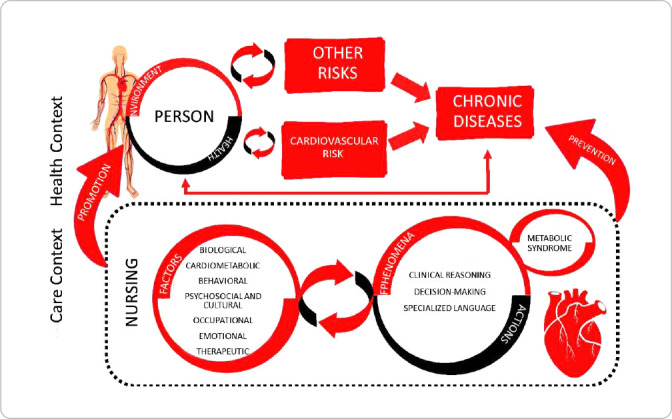
Modeling the concepts of Theory of Care in the Context of Cardiovascular Risk nursing metaparadigm, central and factorial concepts, João Pessoa, Paraíba, Brazil, 2022

It is noteworthy that nursing care must follow the nursing process stages and aims to provide care to healthy people in a context of cardiovascular risk, regardless of the occurrence of related illnesses, assistance in cardiovascular self-care and helping people achieve their human potential. Figure 1 represents, through modeling, how cardiovascular risk is related to a person’s health and care context, the environment, health and nursing.

**Figure 2:**
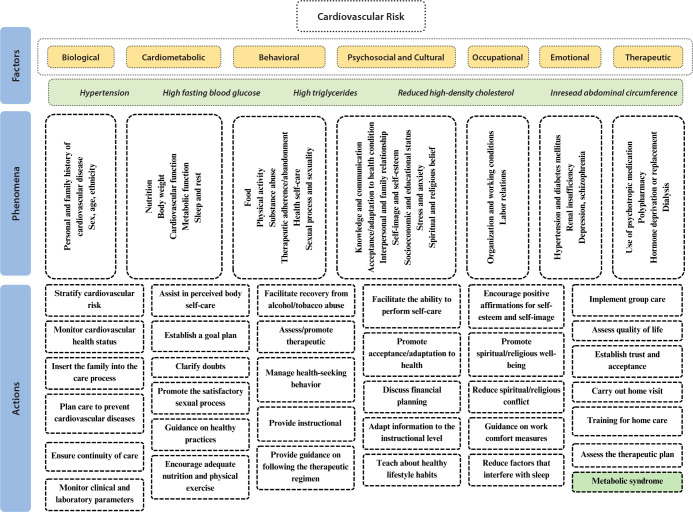
Modeling the Theory of Care in the Context of Cardiovascular Risk factorial concepts, João Pessoa, Paraíba, Brazil, 2022

The layout of theoretical modeling for nursing care in the context of cardiovascular risk considers factorial concepts, phenomena and nursing actions as a multidirectional and interrelated open system to promote health and prevent cardiovascular diseases. In TEORISC, the scope of care in the context of cardiovascular risk involves: identification of concepts related to nursing domain to identify the central phenomenon and insert a person in the best conditions for nature to restore/maintain their health by reducing risk; promotion of adaptation and care by a person to the risk condition; development of an interaction between a nursing professional and a person so that care promotes health through harmony with the environment. To this end, nursing actions are necessary for factorial concepts in the context of cardiovascular risk, as shown in Figure 2.

TEORISC propositions are fundamental for a broad understanding of the context of cardiovascular risk, but are not limited to them, and may be useful for other disciplines in which researchers and nursing and healthcare professionals must apply, replicate and test the propositions and their relationships to consolidate the knowledge initially proposed, but like all theories, they are subject to criticism, preferably constructive and integrative.

TEORISC provides a theoretical contribution to understanding cardiovascular risk as a context of life with vulnerability related to modifiable and non-modifiable, significant and interrelated factors that compromise a person’s health and that require nursing and healthcare at all levels of life. By bringing together relevant phenomena for identifying cardiovascular risk, metabolic syndrome is considered the central and circumscribed phenomenon linked to factors and other phenomena that, once aggregated, are added to increase cardiovascular risk and the impact at the individual, collective, social and political levels.

Cardiovascular risk factors are multiple, contribute to increased risk and morbidity and mortality and need to be recognized in care. These factors can be modifiable or non-modifiable, involving phenomena to be analyzed in terms of their spectrum, and are included in TEORISC as conditioning and/or associated factorial concepts composed of specific phenomena to be identified during care.

The relevance of nursing classification systems stands out, especially ICNP^®^ terminological subset, as the conceptual basis of this study. Through this, it will be possible to record the nursing process using a professional language and a theory that represents the discipline and its role in the area, with the potential to generate health indicators that are still perceived in an abstract and/or generalized way by health indicators from other disciplines.

### Theory assumptions

TEORISC is made up of 16 assumptions related to care in the context of cardiovascular risk, as described below:

1. Cardiovascular risk precedes people’s cardiovascular disease, and its correct identification can prevent or postpone the development of chronic diseases;
2. Cardiovascular risk factors are varied, conditioning and associated, multidimensional and, for the most part, precede metabolic syndrome;
3. If metabolic syndrome is the central phenomenon in the context of cardiovascular risk, this will be the most frequent phenomenon in people;
4. Cardiovascular risk may be greater or equal when a person presents fewer than three criteria for metabolic syndrome, added to other conditioning and associated risk factors, not directly related to one of the five criteria for the syndrome;
5. Cardiovascular risk is the health context and is not necessarily determined by the occurrence of diseases themselves, but rather by risk situations linked or not to comorbidities of cardiovascular etiology;
6. Cardiovascular risk is an open and multidirectional care context, and includes a group of relevant concepts for identifying and prescribing nursing and health actions to reduce risk and morbidity and mortality from chronic diseases;
7. Cardiovascular risk factors involve conditioning factors (cardiometabolic, behavioral and affective) and associated factors (biological, psychosocial, cultural, work and therapeutic), which, when combined, can increase a person’s cardiovascular vulnerability;
8. The identification of the phenomena included in the factors and the implementation of the respective actions are more effective when considering the particularities related to the person, the family, the community and the health and care environments;
9. Cardiovascular risk stratification indicates the risk of developing cardiovascular diseases, and not the chance of dying from this cause in the medium and long term;
10. Cardiovascular risk stratification that considers the conditioning and association of biological, cardiometabolic, behavioral, psychosocial, cultural, occupational, emotional and therapeutic factors is more effective in determining the risk of developing cardiovascular diseases;
11. Vulnerable population groups may have increased cardiovascular risk or a greater number of risk factors, depending on exposure to biological, cardiometabolic, behavioral, psychosocial, cultural, occupational, emotional and therapeutic phenomena;
12. To reduce cardiovascular risk, nursing and healthcare guided by TEORISC is essential;
13. Care programs for managing cardiovascular risk and metabolic syndrome led by nurses and using the nursing process contribute to reducing cardiovascular risk and the rate of morbidity and mortality due to chronic diseases, especially cardiovascular diseases and diabetes *mellitus*;
14. If nursing phenomena (nursing diagnoses, outcomes and interventions), evidenced in nurses’ clinical care, are identified, prescribed and implemented correctly, there will be a reduction in cardiovascular risk in the medium and long term;
15. Nursing interventions for people at cardiovascular risk should facilitate planning, identification of promotion actions, guidance and evaluation of cardiovascular care and self-care;
16. Nursing and healthcare focused on reducing cardiovascular risk considers the relevance of a balanced diet and physical exercise, but is not limited to these, focusing on a person’s individualities and including the family/collectivities and the environment in the process.

## DISCUSSION

MRT was developed through induction of research and practice, originating from a doctoral thesis in nursing, at the *Universidade Federal da Paraíba*, in northeastern Brazil, with emphasis on innovation in the field of disciplinary development through research in Brazil, with potential for global use. The literature states that creating theories tends to consider research results to produce generalizing conclusions^(18)^, especially through doctoral theses^(19)^, and that induction can be used to create theories that are minimally or not derived from already designed models, basically using data to theorize^(20)^, a perspective that converges with the proposal of this study and its final product: TEORISC.

However, authors^(21)^ state that there has not been recognition of the potential to produce theories of theses in Brazilian nursing, especially those guided by inductive reasoning. This study follows the flow contrary to this “invisibility” and presents an MRT that links its concepts, presenting propositions and assumptions schematized in a general and specific modeling regarding the context of cardiovascular risk.

As a practical discipline, it is clear that nursing theory is closely linked to practice, constituting an induction of practice, even when not explicitly referred to^(3)^. Thus, TEORISC was developed through concepts related to research and practice, forming part of an ICNP^®^ terminological subset for the central phenomenon, analyzed and identified in the clinic through the nursing process. The innovative and pioneering aspect regarding MRT development stands out, using ICNP^®^ in all its parts as a conceptual basis.

TEORISC’s philosophical influence on nursing frameworks is highlighted with regard to the nursing process, basic human needs^(15)^ and nursing metaparadigm^(16)^. Even dating back more than 30 years, constructs are contemporary, relevant and contributed strongly to constructing MRT propositions.

Interrelating with metaparadigm concepts as organizers of other concepts that are in the nursing knowledge domain, those that concern the interaction of people with the environment and that can be influenced by nursing acts stand out, which aim to achieve the best possible health of a person being cared for. Therefore, these concepts refer to people’s responses to the context of life and health (diagnoses) and actions that nursing implements to positively influence these responses (interventions), in order to obtain or maintain the best possible health and well-being conditions (outcomes) of a person^(22)^ and community.

In the scenario of producing knowledge specific to nursing, a conceptual model^(5)^ and MRT^(23)^ were constructed for the disciplinary expansion involving cardiovascular risk. However, there are distinctions in relation to TEORISC. The conceptual model^(5)^ presents a structure for approaching people with hypertension, with the hypothesis of the existence of causal relationships between individual and socio-environmental factors that predispose, facilitate and reinforce health behaviors related to the disease, which, in turn, have a causal relationship with the development and progression of cardiovascular risk factors. Unlike TEORISC, this conceptual structure is specifically related to the therapeutic follow-up of a person who has a chronic disease, such as cardiovascular diseases, involving the risk directly evidenced in people with the specific disease without clearly presenting the model propositions and assumptions.

The MRT of culturally sensitive risk perception, with Irish people at risk for cardiovascular diseases and diabetes^(23)^, uses three methodological development strategies (literature review study, qualitative and quantitative), which were also developed in this study. The main concepts of the aforementioned MRT include attention to risk, assessment processes, cognition and affect, different and more synthetic concepts in relation to TEORISC concepts.

MRTs^(23)^ present concepts and statements relevant to risk perception, at the time, linked to people at high risk for cardiovascular diseases, but which can extrapolate this affective aspect and be applied in other contexts that predispose a person to culturally sensitive risks, such as endocrine, neoplastic and infectious diseases. TEORISC focuses on the risk of cardiovascular diseases and increases the body of knowledge in the area by proposing an approach of actions/interventions to be developed in health and nursing practice and research to reduce conditioning and associated risk factors.

TEORISC’s conceptual basis comes from specialty language, aligned with a discipline classification system, analyzed by experts in the field and applied to a population group and a synthesis strategy not used in theories related to cardiovascular risk evidenced in the literature^(5,23)^. It is considered that the strategy used in this study provided a more comprehensive understanding of the context of cardiovascular risk, and can be used by healthcare professionals, especially nurses, with a view to reducing morbidity and mortality from cardiovascular diseases and diabetes *mellitus* as well as their complications.

TEORISC’s cardiovascular risk factors involve aspects of cardiovascular health that are relevant to clinical practice and nursing and health research, varying according to the particularities of a person and the care environment, not limited to those described. Nursing actions are open nursing conduct prescriptions, but must be labeled using a classification system and implemented to reduce a person’s cardiovascular risk, including family and community.

The conceptual model^(5)^ presents planning protocol for health education interventions related to clinical expression of the condition and health-related quality of life. Meanwhile, MRT^(23)^ highlights that nursing interventions guided by an MRT can improve behavior in vulnerable populations. However, both do not mention the use of a concept development model, nursing theory, nursing process or nursing classification system, even in their construction, which essentially integrates TEORISC’s conceptual basis.

The theory represents relationships between the theory concepts as simple, but as comprehensive as possible, emphasizing the theorist’s creative nature^(3)^. Thus, modeling expresses theoretical creativity and facilitates the understanding and application of TEORISC, mainly regarding the relationships between concepts and the insertion of nursing interventions for each specific phenomenon presented by a person and community.

TEORISC is part of research on the state of the art on nursing theory production^(24)^ and technological development^(25)^ through ICNP^®^ as well as partial use of concepts, modeling, propositions and assumptions in various scenarios^(26-28)^, strengthening the research carried out and published by *Stricto Sensu* Graduate Programs, especially in Brazil. By testing and developing criticisms for the advancement of TEORISC, professionals will, in fact, be contributing to refining the theory, providing evidence for health and nursing care, in accordance with the conceptual bases of the discipline.

### Study limitations

Among the limitations, there is the empirical testing of TEORISC components nationally and internationally, which can be carried out by studies with robust and varied methodological designs. Considering the recent proposition, future studies should review it and deepen the understanding of the concepts that make up the theory and their relationships as well as the application of TEORISC components in clinical practice with different population groups.

### Contributions to nursing

TEORISC contributes to people at cardiovascular risk, providing nurses with a holistic view of the people under their care. Therefore, risk factors and phenomena associated with context must be considered to implement specific care aligned with a person’s vital demands, not limited to the biological, technical aspects and unlinked to the human factor. With TEORISC constructs, scientific and technological productions, new diagnoses, outcomes and interventions using other classification systems, instruments/scales and innovative elements can be developed and tested to strengthen care in the context of cardiovascular risk.

## FINAL CONSIDERATIONS

An MRT was developed with the aim of describing care in the context of cardiovascular risk, the relationship of conditioning and associated factors and also prescribing actions to promote health and reduce risk to reduce morbidity and mortality due to chronic diseases. The application of concept analysis and theoretical induction strategies based on ICNP^®^ terminological subset demonstrated the potential for building one’s own knowledge arising from the nursing process, generating propositions, assumptions and modeling, which will enable theoretical testing. The originality, novelty and innovation stand out for pragmatism in care to reduce morbidity and mortality from cardiovascular diseases in the medium and long term of the world population.
