# Supplementary material for: Middle-range theory for nursing for care in the context of cardiovascular risk
Source: Rev Bras Enferm. 2024 Sep 20;77(4):e20240190. doi: 10.1590/0034-7167-2024-0190 (PMC11419686; doi:10.1590/0034-7167-2024-0190)
Supplement: Translation

# Translation: Teoria de médio alcance de enfermagem para o cuidado no contexto de risco cardiovascular

**DOI:** 10.1590/0034-7167-2024-0190pt

**Authors:** Nuno Damácio de Carvalho Félix, Alba Lúcia Bottura Leite de Barros, Maria Miriam Lima da Nóbrega

**Affiliations:** I Universidade Federal do Recôncavo da Bahia. Santo Antônio de Jesus, Bahia, Brasil; II Universidade Federal de São Paulo. São Paulo, São Paulo, Brasil; III Universidade Federal da Paraíba. João Pessoa, Paraíba, Brasil

**Keywords:** Teoria de Enfermagem, Modelos Teóricos, Fatores de Risco de Doenças Cardíacas, Formação de Conceito, Terminologia Padronizada em Enfermagem.

## Abstract

**Objetivos:**

desenvolver uma teoria de médio alcance de enfermagem para o cuidado no contexto de risco cardiovascular.

**Métodos:**

estudo de desenvolvimento teórico, por meio da indução pela pesquisa e pelos padrões da prática da CIPE^®^, realizado em seis etapas: análise de conceitos; estruturação do subconjunto terminológico da CIPE^®^; contextualização e finalidade da teoria; construção das proposições; modelagem; e construção dos pressupostos.

**Resultados:**

a Teoria do Cuidado no Contexto de Risco Cardiovascular possui âmbito de médio alcance, descrevendo o cuidado, prescrevendo ações para promover a saúde e a redução do risco cardiovascular. Foram construídas 13 proposições em três categorias (conceitos do metaparadigma da enfermagem, centrais e fatoriais), duas modelagens e 16 pressupostos.

**Considerações Finais:**

a teoria contribui com a construção de conhecimento próprio oriundo do processo de enfermagem para o cuidado no contexto de risco cardiovascular, gerando proposições, pressupostos e modelagem, as quais possibilitarão testagem teórica.

## INTRODUÇÃO

A população mundial, incluindo o Brasil, passou por processos de transformação socioeconômica e epidemiológica no século passado. A inserção de inúmeras tecnologias advindas da modernização favoreceu a melhoria da qualidade de vida das pessoas, pela maior comodidade no cotidiano, mas também afetou os hábitos relacionados ao risco cardiovascular, os quais influenciam de maneira crucial o processo saúde-doença^(1)^ e o conhecimento desenvolvido sobre o cuidado dessas pessoas. Junto a esses processos, as disciplinas profissionais, incluindo a enfermagem, têm buscado se desenvolver na perspectiva de acompanhar os avanços, assim como melhorar a assistência e a comunicação em saúde, para reduzir os impactos desses progressos, formulando novas políticas e teorias no campo da saúde.

A prática clínica de enfermagem demanda o desenvolvimento do conhecimento próprio, em especial sobre o cuidado de pessoas com risco cardiovascular, tendo em vista que implicam vulnerabilidade, que contribui para o surgimento de doenças e complicações, muitas vezes, irreversíveis. Destaca-se a atuação fundamental da equipe multiprofissional, em especial do enfermeiro, a fim de identificar, intervir, coibir e produzir conhecimento sobre os fatores de risco cardiovascular^(2)^.

Tem-se a necessidade de investigações para descrever o cuidado às pessoas com risco cardiovascular baseado em evidências e fundamentados em teorias de enfermagem direcionadas e induzidas da prática clínica, com conceitos definidos e vinculados ao processo de enfermagem e aos padrões da prática, utilizando sistemas de classificação de enfermagem. Nesse contexto, as teorias de médio alcance (TMAs) são parte da base de conhecimento da enfermagem para orientar a prática e pesquisa, e seu uso contínuo oferece um potencial para testar e evoluir o conhecimento da enfermagem^(3)^.

O desenvolvimento de uma TMA envolve a análise crítica do corpo de conhecimento produzido, que precisa ser organizado e descrito para subsidiar o cuidado de enfermagem à população específica; no caso deste estudo, tem-se as pessoas com risco cardiovascular. O cuidado de enfermagem fundamentado em uma teoria própria contribui para a construção da ciência e disciplina de enfermagem, pois as bases teóricas de uma ciência advêm de um processo dinâmico e criativo, desenvolvido para explicar e descrever os elementos relacionados à prática^(4)^. De tal modo, considerando a magnitude das doenças cardiovasculares, sobretudo no contexto brasileiro, é importante que o enfermeiro acesse e utilize um suporte teórico-conceitual da disciplina que permita, de forma racional e objetiva, o planejamento e a avaliação de intervenções para o controle dos fatores de risco^(5)^, impactando a saúde cardiovascular da população.

As TMA envolvem fenômenos relativamente concretos e específicos, declarando o que eles são, por que ocorrem e como ocorrem, fornecendo suporte ao entendimento quanto ao comportamento, situações, eventos e conexão entre os fenômenos de enfermagem (diagnósticos, resultados e intervenções de enfermagem)^(6)^. Apresenta-se o processo de desenvolvimento de uma TMA por indução dos padrões da prática e da pesquisa de enfermagem estruturados no subconjunto terminológico da Classificação Internacional para a Prática de Enfermagem (CIPE^®^).

Especificamente quanto ao desenvolvimento de TMA por meio de padrões da prática, tem-se como função descrever, explicar, ou prever os fenômenos de forma explícita e testável, diferentemente das grandes teorias^(7)^. Entretanto, evidenciam-se a escassez de estudos com esse delineamento e as evidências existentes, ainda pulverizadas, com falhas na estrutura de conexão explicativa ou descritiva sobre o risco cardiovascular, seus fatores e cuidados necessários.

O desenvolvimento da TMA utilizando um sistema de classificação de enfermagem expressa a disciplina, com a compreensão intersubjetiva do significado dos conceitos incluídos na linguagem profissional, e a organização dos conceitos que a constituem em um universo comum de percepção e de comunicação da enfermagem^(8)^. Assim, o presente estudo pode contribuir com a construção do corpo de conhecimento da enfermagem, com os componentes descritos e marco teórico-conceitual concreto, factível e testável no cuidado direcionado às pessoas com risco cardiovascular.

## OBJETIVOS

Desenvolver uma TMA de enfermagem para o cuidado no contexto de risco cardiovascular.

## MÉTODO

### Aspectos éticos

Por se tratar de estudo teórico, dispensou-se a submissão ao Comitê de Ética em Pesquisa, mas foram considerados os princípios da pesquisa com seres humanos, por induzir a TMA da prática e da pesquisa.

### Tipo do estudo

Trata-se de estudo de desenvolvimento teórico, orientado para elaboração de uma TMA, utilizando como estratégia a indução pela pesquisa e pelos padrões da prática oriundos do subconjunto terminológico da CIPE^®^.

### Procedimentos metodológicos

A indução da teoria ocorreu por meio de seis etapas: 1) análise de conceitos; 2) estruturação do subconjunto terminológico da CIPE^®^; 3) contextualização do desenvolvimento teórico e a sua finalidade; 4) construção das proposições; 5) modelagem da teoria; e 6) construção dos pressupostos teóricos. Destaca-se a inovação quanto ao método desenvolvido neste estudo, com limitado acervo literário sobre o processo de indução de TMA a partir da CIPE^®^.

Inicialmente, as etapas de análise de conceito^(9-10)^ e estruturação do subconjunto terminológico da CIPE^®(11-13)^ forneceram subsídio para a indução inicial da TMA. A contextualização e a descrição da finalidade da TMA explicitaram o desenvolvimento, relacionando-o ao contexto específico do cuidado de enfermagem, sociopolítico e clínico-epidemiológico, de forma descritiva, vinculado às proposições não relacionais e aos pressupostos alinhados ao fenômeno principal no contexto de risco cardiovascular, síndrome metabólica, justificando-se a utilização da análise desse conceito como primeira base empírica da teoria.

Quanto à construção das proposições da TMA, tem-se a relação entre dois ou mais conceitos, e são usados para conectar conceitos na criação de teorias antes da realização de explanações ou previsões^(7)^. Essas podem ser não relacionais, correspondendo às definições dos conceitos, declarando algum tipo de relação entre dois ou mais conceitos e podendo ter uma relação de associação ou casualidade^(14)^. Considerando a estratégia dessa etapa, apresentou-se a definição dos conceitos principais (risco cardiovascular e síndrome metabólica) e dos conceitos fatoriais condicionantes e associados ao risco cardiovascular, correlacionados aos conceitos do metaparadigma da enfermagem, com influência filosófica de referenciais da disciplina^(15-16)^.

A etapa de modelagem da teoria envolve representação esquemática das relações entre os conceitos da TMA, apresentando aspectos da realidade sobre os fenômenos da TMA por meios variados, como formas geométricas e diagramas. Neste estudo, foram realizadas duas modelagens teóricas que representam a visão de mundo real dos fenômenos da TMA com uso da linguagem ou símbolos e setas direcionais^(7)^.

Nas modelagens, considerou-se a produção intelectual e reflexiva sobre os fenômenos da TMA oriundos das etapas anteriores, com conceitos apresentados em uma estrutura imagética coerente com as definições, proposições e pressupostos construídos, utilizando a Norma NBR 8403^(17)^ para o escalonamento de linhas para uso em desenhos técnicos e documentos semelhantes, com apoio de *design* gráfico com formação em enfermagem e *expertise* na área da enfermagem cardiovascular.

Os pressupostos envolvem asserções factuais conhecidas pela experiência ou podem refletir posições de valor que implicam o que está certo, o que é bom ou deveria ser^(14)^. Nessa perspectiva, foram construídos os pressupostos da TMA por meio do corpo de conhecimento analisado e estruturado pelas etapas anteriores^(9-13)^, os quais subsidiam a testagem da TMA.

## RESULTADOS

### Contextualização da teoria

A Teoria do Cuidado no Contexto de Risco Cardiovascular, denominada, pelos autores, como TEORISC, possui âmbito de TMA de enfermagem, do tipo descritiva, e foi desenvolvida por meio de vários estudos, vinculados entre si e articulados para a identificação, análise conceitual e de conteúdo, estruturação e aplicação clínica de um subconjunto terminológico da CIPE^®^, em que todos os componentes foram induzidos da pesquisa e dos padrões da prática a partir do processo de enfermagem.

A TEORISC é inserida como alicerce teórico para orientar o cuidado de enfermagem e saúde em um momento em que se tem elevadas taxas de morbimortalidade por doenças crônicas não transmissíveis, em especial as doenças cardiovasculares e diabetes *mellitus*, situações que podem ser preveníveis com a mudança de hábitos e a redução dos fatores de risco. A teoria contribui para o avanço no conhecimento sobre o cuidado direcionado ao contexto de risco cardiovascular, ao apresentar aspectos relevantes sobre as ações do enfermeiro no processo de redução dos fatores de risco em momento sociopolítico que demanda a elevação da visibilidade da disciplina pela consolidação do corpo de conhecimento e geração de indicadores de saúde relacionados à prática de enfermagem.

Soma-se à produção de conhecimento oriunda de um fenômeno central - síndrome metabólica - em um contexto de saúde - risco cardiovascular -, com a organização e descrição dos fatores e fenômenos relevantes, direta ou indiretamente. As ações de enfermagem integrantes da TEORISC são vinculadas a um sistema de classificação que contempla diagnósticos, resultados e intervenções de enfermagem que está em processo de aprimoramento constante nos âmbitos nacional e internacional: a CIPE^®^.

Os componentes da TEORISC estão apresentados, definidos e representados em modelagens que facilitam a compreensão dos conhecimentos produzidos. Os conceitos e suas relações, proposições e pressupostos estão discutidos quanto à relação com a realidade e o conhecimento existente, seu impacto nos serviços de saúde e a indicação para uso na prática e na pesquisa, a fim de promover o teste e a geração de evidências quanto ao impacto da TEORISC na saúde cardiovascular nas variadas populações do Brasil e do mundo.

### Finalidades da teoria

A TEORISC descreve o cuidado no contexto de risco cardiovascular, a relação dos fatores de risco cardiovascular condicionantes e associados que antecedem e aumentam a vulnerabilidade da pessoa. Tem-se, também, a prescrição de ações para promover a saúde e a redução do risco cardiovascular alinhado aos fatores e aos fenômenos individuais para reduzir a morbimortalidade por doenças crônicas não transmissíveis a médio e longo prazo.

### Proposições e modelagem da teoria

A TEORISC é composta por 13 proposições, com suas respectivas definições, organizadas em três categorias envolvendo os conceitos do metaparadigma da enfermagem, os conceitos centrais e fatoriais (condicionantes e associados) ao cuidado no contexto de risco cardiovascular, conforme descrito no Quadro 1.

**Quadro 1:** Proposições da Teoria do Cuidado no Contexto de Risco Cardiovascular, João Pessoa, Paraíba, Brasil, 2022

Proposições da TEORISC	
Conceitos	Definições
Metaparadigma da enfermagem	
Pessoa	Ser humano holístico, aberto, integrado, adaptável e complexo em um contexto de risco cardiovascular no seu ambiente/contexto de saúde e que apresenta necessidades humanas e sociais (físicas, bioquímicas, intelectuais, psicossociais e culturais).
Saúde	Unidade mente, corpo e alma, com funcionalidade independente, adaptada e com sua melhor condição de saúde diante dos estressores cardiovasculares, intrínsecos e extrínsecos e outros riscos no contexto de saúde.
Ambiente	Elementos internos e externos, concretos e abstratos de um sistema aberto que afetam a pessoa, seu organismo e o contexto de cuidado e de saúde.
Enfermagem	Ciência, arte e disciplina prática que envolve o cuidado utilizando fundamentos científicos na construção do marco teórico-prático. Identifica contextos, fatores e fenômenos relevantes para a saúde cardiovascular por meio do uso do raciocínio clínico no processo de tomada de decisão.
Conceitos centrais	
Risco cardiovascular	Contexto de saúde e de cuidado que permite a identificação de grupos com fatores de risco para doenças cardiovasculares, modificáveis (cardiometabólicos, comportamentais, psicossociais e culturais, laborais, afecciosos e terapêuticos) e não modificáveis (biológicos), que atuam como marcadores precoces e inter-relacionados, de etiologia múltipla e heterogênica, que predispõem a pessoa e sua coletividade à vulnerabilidade.
Síndrome metabólica	Agregação de marcadores de risco cardiovasculares significativos, de etiologia multifatorial, relacionada à inflamação assintomática que predispõe a pessoa à vulnerabilidade. Envolve a identificação de, pelo menos, três critérios diagnósticos, como aumento da circunferência abdominal, elevação da glicemia vascular de jejum, da pressão arterial, dos triglicerídeos, e/ou redução do colesterol de alta densidade, de acordo com o parâmetro adotado e a demanda de abordagem multidisciplinar, nela inserida a enfermagem.
Conceitos fatoriais	
Biológicos	Fatores não modificáveis relacionados à vida intrínseca da pessoa, inseridos pelo tempo de existência, genética e/ou hereditariedade. Envolvem fenômenos como sexo/gênero, idade, etnia, histórico familiar.
Cardiometabólicos	Fatores relacionados à função cardiovascular, ao processo hormonal e de nutrição, modificáveis a depender das características específicas dos fenômenos de risco, com impacto na saúde da pessoa. Envolvem fenômenos como nutrição, peso corporal, concentração de estrógeno e progesterona, menopausa, sono e repouso.
Comportamentais	Fatores modificáveis relacionados à maneira, individual e/ou coletiva, de proceder frente a estímulos sociais e/ou afetuosos que comprometem a saúde e/ou cuidado cardiovascular. Envolvem fenômenos como hábitos alimentares, atividade/exercício físico, uso de tabaco/álcool, baixa adesão, abandono terapêutico, manejo e autocuidado em saúde, processo sexual e sexualidade.
Psicossociais e culturais	Fatores que envolvem aspectos psicológicos, sociais e culturais, modificáveis a depender do interesse, disposição, contexto coletivo, autopercepção ou crença pessoal, que em conjunto comprometem a saúde e/ou cuidado cardiovascular. Envolvem fenômenos como conhecimento, comunicação, aceitação e adaptação à condição de saúde, relações interpessoais e familiares, autoimagem, autoestima, condição socioeconômica e cultural, estresse, ansiedade, crença espiritual e religiosa.
Laborais	Fatores modificáveis relacionados ao processo de trabalho, sua organização e aos problemas decorrentes da exaustão ocupacional da pessoa, que comprometem a saúde e/ou cuidado cardiovascular.
Afecciosos	Fatores relacionados a doenças específicas e/ou impacto no corpo da pessoa, modificáveis a depender da disponibilidade de tratamento para cura, que comprometem a saúde e/ou cuidado cardiovascular.
Terapêuticos	Fatores relacionados a processos, tecnologias e procedimentos para tratar doenças da pessoa que comprometem a saúde e/ou cuidado cardiovascular, modificáveis por meio da intervenção no processo terapêutico ou cessação de falhas e/ou erros na condução. Envolvem fenômenos como o uso de medicamentos específicos, polifarmácia, privação ou reposição hormonal ou tratamentos especiais.

Na TEORISC, a pessoa está relacionada ao ser humano com fatores de risco cardiovascular condicionantes para o desenvolvimento do adoecimento (cardiometabólicos, comportamentais e afecciosos) e associados entre si ou com outros fatores (biológicos, psicossociais e culturais, laborais e terapêuticos), que demandam cuidados para prevenção de doenças crônicas não transmissíveis, promoção da saúde e manutenção da vida saudável, identificados pela evidência do fenômeno central. A saúde deve ser o propósito do cuidado no contexto de risco cardiovascular para evitar, controlar ou eliminar o comprometimento do fenômeno central e/ou promover saúde e prevenir doenças cardiovasculares.

No ambiente, insere-se a enfermagem, com vistas a reduzir/eliminar o risco cardiovascular e seu fenômeno central, utilizando uma linguagem padronizada, prescrevendo ações de enfermagem para redução do risco cardiovascular, eliminação do fenômeno central e promoção da saúde e prevenção de doenças cardiovasculares e demais doenças crônicas não transmissíveis.

Destaca-se que o cuidado de enfermagem deve seguir as etapas do processo de enfermagem e tem como metas o atendimento da pessoa saudável em contexto de risco cardiovascular, independentemente da ocorrência de enfermidades correlatas, a assistência no autocuidado cardiovascular e o auxílio à pessoa para atingir seu potencial humano. A Figura 1 representa, por meio da modelagem, como o risco cardiovascular está relacionado ao contexto de saúde e de cuidado da pessoa, ao ambiente, à saúde e à enfermagem.

**Figura 1:** Modelagem dos conceitos do metaparadigma da enfermagem, conceitos centrais e fatoriais da Teoria do Cuidado no Contexto de Risco Cardiovascular, João Pessoa, Paraíba, Brasil, 2022

A diagramação da modelagem teórica para o cuidado de enfermagem no contexto de risco cardiovascular considera os conceitos fatoriais, os fenômenos e as ações de enfermagem um sistema aberto multidirecional e inter-relacionado para promover a saúde e prevenir doenças cardiovasculares. Na TEORISC, o escopo do cuidado no contexto de risco cardiovascular envolve: identificação dos conceitos relacionados ao domínio da enfermagem para identificação do fenômeno central e inserção da pessoa nas melhores condições para que a natureza restaure/mantenha sua saúde pela redução do risco; promoção da adaptação e do cuidado pela pessoa à condição de risco; desenvolvimento de uma interação entre o profissional de enfermagem e a pessoa para que o cuidado promova a saúde pela harmonia com o ambiente. Para tal, são necessárias ações de enfermagem para conceitos fatoriais do contexto de risco cardiovascular, conforme apresentados na Figura 2.

**Figura 2:** Modelagem dos conceitos fatoriais da Teoria do Cuidado no Contexto de Risco Cardiovascular, João Pessoa, Paraíba, Brasil, 2022

As proposições da TEORISC são fundamentais para a compreensão ampla do contexto de risco cardiovascular, mas não se limitam aos mesmos, podendo ser úteis para outras disciplinas em que pesquisadores e profissionais de enfermagem e saúde devem aplicar, replicar e testar as proposições e suas relações para consolidar o conhecimento inicialmente proposto, mas como toda teoria, está sujeita a críticas, preferencialmente construtivas e integradoras.

A TEORISC contempla um aporte teórico para a compreensão do risco cardiovascular como um contexto de vida com vulnerabilidade relacionada a fatores modificáveis e não modificáveis, significativos e inter-relacionados, que comprometem a saúde da pessoa e que demandam cuidado de enfermagem e saúde em todos os níveis da vida. Por agregar fenômenos relevantes para identificação do risco cardiovascular, a síndrome metabólica é considerada o fenômeno central e circunscrito vinculado aos fatores e demais fenômenos que, uma vez agregados, são somados para elevar o risco cardiovascular e o impacto no nível individual, coletivo, social e político.

Os fatores de risco cardiovascular são múltiplos, contribuem para a elevação do risco e da morbimortalidade e precisam ser reconhecidos no cuidado. Esses fatores podem ser modificáveis ou não modificáveis, envolvendo fenômenos a serem analisados quanto ao seu espectro, e estão inseridos na TEORISC como conceitos fatoriais condicionantes e/ou associados compostos por fenômenos específicos a serem identificados durante o cuidado.

Destaca-se a relevância dos sistemas de classificação em enfermagem, sobretudo o subconjunto terminológico da CIPE^®^, como base conceitual deste estudo. Por meio deste, será possível registrar o processo de enfermagem utilizando uma linguagem profissional e uma teoria que represente a disciplina e seu papel na área, com potencialidade para gerar indicadores de saúde que ainda são percebidos de forma abstrata e/ou generalizada por indicadores de saúde de outras disciplinas.

### Pressupostos da teoria

A TEORISC é composta por 16 pressupostos, relacionados ao cuidado no contexto de risco cardiovascular, conforme descrito a seguir:

1. O risco cardiovascular antecede o adoecimento cardiovascular da pessoa, e sua correta identificação pode prevenir ou postergar o desenvolvimento de doenças crônicas;
2. Os fatores de risco cardiovascular são variados, condicionantes e associados, multidimensionais e, em sua maioria, antecedem a síndrome metabólica;
3. Se síndrome metabólica é o fenômeno central no contexto de risco cardiovascular, esse será o fenômeno mais frequente nas pessoas;
4. O risco cardiovascular pode ser maior ou igual quando a pessoa apresenta menos que três critérios da síndrome metabólica, somados a outros fatores de risco condicionantes e associados, não diretamente relacionados a um dos cinco critérios da síndrome;
5. O risco cardiovascular é o contexto de saúde e não é, obrigatoriamente, determinado pela ocorrência de doenças propriamente ditas, mas sim a situações de risco vinculadas ou não às comorbidades de etiologia cardiovascular;
6. O risco cardiovascular é um contexto de cuidado, aberto e multidirecional, e contempla um grupo de conceitos relevantes para identificação e prescrição de ações de enfermagem e saúde para a redução do risco e da morbimortalidade por doenças crônicas;
7. Os fatores de risco cardiovascular envolvem fatores condicionantes (cardiometabólicos, comportamentais e afecciosos) e associados (biológicos, psicossociais, culturais, laborais e terapêuticos) que, quando agregados, podem elevar a vulnerabilidade cardiovascular da pessoa;
8. A identificação dos fenômenos inseridos nos fatores e a implementação das respectivas ações são mais efetivas quando consideram as particularidades relacionadas à pessoa, à família, à coletividade e ao ambiente de saúde e de cuidado;
9. A estratificação do risco cardiovascular sinaliza o risco de desenvolver doenças cardiovasculares, e não a chance de morrer por essa causa a médio e longo prazo;
10. A estratificação do risco cardiovascular que considera o condicionamento e associação dos fatores biológicos, cardiometabólicos, comportamentais, psicossociais, culturais, laborais, afecciosos e terapêuticos é mais efetiva na determinação do risco de desenvolver doenças cardiovasculares;
11. Grupos populacionais vulnerabilizados podem ter risco cardiovascular aumentado ou maior número de fatores de risco, a depender da exposição aos fenômenos biológicos, cardiometabólicos, comportamentais, psicossociais, culturais, laborais, afecciosos e terapêuticos;
12. Para redução do risco cardiovascular, é fundamental o cuidado de enfermagem e saúde guiado pela TEORISC;
13. Programas de cuidado para manejo do risco cardiovascular e da síndrome metabólica conduzidos por enfermeiros e utilizando o processo de enfermagem contribuem para a redução do risco cardiovascular e da taxa de morbimortalidade por doenças crônicas, em especial as doenças cardiovasculares e diabetes *mellitus*;
14. Se os fenômenos de enfermagem (diagnósticos, resultados e intervenções de enfermagem), evidenciados no cuidado clínico do enfermeiro, forem identificados, prescritos e implementados corretamente, haverá redução do risco cardiovascular em médio e longo prazo;
15. As intervenções de enfermagem para pessoas com risco cardiovascular devem facilitar o planejamento, a identificação das ações de promoção, orientação e a avaliação do cuidado e autocuidado cardiovascular;
16. O cuidado de enfermagem e saúde com foco na redução do risco cardiovascular considera a relevância da alimentação equilibrada e da prática de exercício físico, mas não se limita a esses, centrando-se nas individualidades da pessoa e inserindo a família/coletividade e o ambiente no processo.

## DISCUSSÃO

Desenvolveu-se a TMA por indução da pesquisa e da prática, proveniente de uma tese de doutorado em enfermagem, na Universidade Federal da Paraíba, no nordeste brasileiro, com destaque para a inovação no campo do desenvolvimento disciplinar por meio de pesquisas do Brasil, com potencial de uso global. A literatura refere que a criação de teorias tende a considerar resultados de pesquisa para produzir as conclusões generalizantes^(18)^, em especial por meio de teses de doutorado^(19)^, e que a indução pode ser utilizada para criar teorias minimamente ou não derivadas de modelos já concebidos, usando basicamente os dados para teorizar^(20)^, perspectiva que converge com a proposta deste estudo e seu produto final: a TEORISC.

No entanto, autores^(21)^ afirmam que não se tem verificado o reconhecimento da potencialidade produtora de teorias das teses na enfermagem brasileira, principalmente as orientadas por raciocínio indutivo. Este estudo segue o fluxo contrário a essa “invisibilidade” e apresenta a TMA que vincula seus conceitos, apresentando proposições e pressupostos esquematizados em uma modelagem geral e específica quanto ao contexto de risco cardiovascular.

Como uma disciplina prática, é evidente que a teoria de enfermagem está intimamente ligada à prática, configurando-se uma indução da prática, mesmo quando não explicitamente referida^(3)^. Assim, a TEORISC foi desenvolvida por meio de conceitos relacionados à pesquisa e à prática, integrantes de um subconjunto terminológico da CIPE^®^ para o fenômeno central, analisados e identificados na clínica por meio do processo de enfermagem. Destaca-se o aspecto inovador e pioneiro quanto ao desenvolvimento de TMA, utilizando como base conceitual a CIPE^®^ em todas as suas partes.

Negrita-se a influência filosófica da TEORISC em referenciais da enfermagem no que tange ao processo de enfermagem, às necessidades humanas básicas^(15)^ e ao metaparadigma da enfermagem^(16)^. Mesmo datados há mais de 30 anos, os construtos são contemporâneos, relevantes e contribuíram fortemente na construção das proposições da TMA.

Inter-relacionando aos conceitos do metaparadigma como organizadores de outros conceitos que estão no domínio de conhecimento da enfermagem, destacam-se aqueles que dizem respeito à interação da pessoa com o ambiente e que podem ser influenciados por atos da enfermagem, que têm como meta a melhor saúde possível da pessoa de quem se está cuidando. Logo, esses conceitos referem-se às respostas das pessoas ao contexto de vida e saúde (diagnósticos) e às ações que a enfermagem implementa para influenciar positivamente essas respostas (intervenções), a fim de obter ou manter as melhores condições possíveis de saúde e bem-estar (resultados) da pessoa^(22)^ e da coletividade.

No cenário da produção de conhecimento próprio da enfermagem, modelo conceitual^(5)^ e TMA^(23)^ foram construídos para a ampliação disciplinar envolvendo o risco cardiovascular. Entretanto, tem-se distinções em relação à TEORISC. O modelo conceitual^(5)^ apresenta estrutura para abordagem da pessoa com hipertensão, com a hipótese da existência de relações causais entre fatores individuais e socioambientais que predispõem, facilitam e reforçam comportamentos em saúde relacionados à doença que, por sua vez, possuem relação causal com o desenvolvimento e progressão dos fatores de risco cardiovascular. Diferente da TEORISC, a referida estrutura conceitual relaciona-se, especificamente, ao seguimento terapêutico da pessoa que apresenta a doença crônica, como as doenças cardiovasculares, envolvendo o risco diretamente evidenciado nas pessoas com a doença específica sem apresentar claramente as proposições e os pressupostos do modelo.

A TMA da percepção de risco culturalmente sensível, com irlandeses com risco para doenças cardiovasculares e diabetes^(23)^, utiliza três estratégias metodológicas de desenvolvimento (estudo de revisão da literatura, qualitativo e quantitativo), as quais também foram desenvolvidas neste estudo. Os principais conceitos da referida TMA incluem atenção ao risco, processos de avaliação, cognição e afeto, conceitos diferentes e mais sintéticos em relação aos conceitos da TEORISC.

A TMA^(23)^ apresenta conceitos e afirmativas relevantes para a percepção de risco, à época, vinculados a pessoas com alto risco para doenças cardiovasculares, mas que podem extrapolar essa vertente afecciosa e ser aplicada em outros contextos que predispõem a pessoa a riscos sensíveis culturalmente, como doenças endócrinas, neoplásicas e infecciosas. A TEORISC concentra-se no risco para doenças cardiovasculares e incrementa o corpo de conhecimento na área, ao propor uma abordagem de ações/intervenções a serem desenvolvidos na prática e na pesquisa em saúde e de enfermagem para redução de fatores de riscos condicionantes e associados.

A base conceitual da TEORISC advém da linguagem de especialidade, alinhado a um sistema de classificação da disciplina, analisado por especialistas na área e aplicado com um grupo populacional e estratégia de síntese não utilizada nas teorias relacionadas ao risco cardiovascular evidenciadas na literatura^(5,23)^. Considera-se que a estratégia utilizada neste estudo proporcionou um entendimento mais abrangente do contexto de risco cardiovascular, podendo ser utilizado por profissionais de saúde, em especial enfermeiros, com vistas a reduzir a morbimortalidade por doenças cardiovasculares e diabetes *mellitus*, assim como suas complicações.

Os fatores de risco cardiovascular da TEORISC envolvem aspectos de saúde cardiovascular que são relevantes para a prática clínica e pesquisa de enfermagem e saúde, variando de acordo com as particularidades da pessoa e do ambiente de cuidado, não se limitando aos descritos. As ações de enfermagem são prescrições de condutas de enfermagem, abertas, mas que devem ser rotuladas utilizando um sistema de classificação e implementadas para a redução do risco cardiovascular da pessoa, nelas inseridas a família e a coletividade.

O modelo conceitual^(5)^ apresenta o protocolo de planejamento para intervenções de educação em saúde relacionadas à expressão clínica da afecção e qualidade de vida relacionada à saúde. Enquanto isso, a TMA^(23)^ destaca que intervenções de enfermagem orientadas por uma TMA podem melhorar o comportamento em populações vulneráveis. Todavia, ambas não fazem menção à utilização de um modelo de desenvolvimento de conceito, teoria de enfermagem, processo de enfermagem ou sistema de classificação em enfermagem, mesmo que na sua construção, o que essencialmente integra a base conceitual da TEORISC.

A teoria representa relações entre os conceitos da teoria como simples, mas o mais abrangente possível, enfatizando a natureza criativa do teórico^(3)^. Assim, as modelagens expressam a criatividade teórica e facilitam o entendimento e aplicação da TEORISC, principalmente quanto às relações entre os conceitos e a inserção de intervenções de enfermagem para cada fenômeno específico apresentado pela pessoa e coletividade.

A TEORISC está inserida em pesquisas sobre o estado da arte sobre a produção de teoria de enfermagem^(24)^ e desenvolvimento tecnológico^(25)^ por meio da CIPE^®^, assim como a utilização em parte dos conceitos, modelagem, proposições e pressupostos em variados cenários^(26-28)^, fortalecendo as pesquisas realizadas e publicadas por Programas de Pós-Graduação *Stricto Sensu*, especialmente no Brasil. Ao testar e desenvolver críticas para o avanço da TEORISC, os profissionais estarão, de fato, contribuindo com o refinamento da teoria, fornecendo evidências para o cuidado de saúde e de enfermagem, de acordo com as bases conceituais da disciplina.

### Limitações do estudo

Entre as limitações, tem-se o teste empírico dos componentes da TEORISC no cenário nacional e internacional, o que poderá ser realizado por estudos com delineamentos metodológicos robustos e variados. Considerando a proposição recente, estudos futuros devem revisá-la e aprofundar a compreensão dos conceitos que compõem a teoria e suas relações, assim como a aplicação dos componentes da TEORISC na prática clínica com variados grupos populacionais.

### Contribuições para área da enfermagem

A TEORISC contribui para pessoas com risco cardiovascular, proporcionando ao enfermeiro uma visão holística das pessoas sob seu cuidado. Assim, deverá considerar os fatores de risco e os fenômenos associados ao contexto para implementação de cuidados específicos e alinhados às demandas vitais da pessoa, não se limitando ao aspecto biológico, tecnicista e desvinculado ao fator humano. Com os construtos da TEORISC, produções científicas e tecnológicas, novos diagnósticos, resultados e intervenções utilizando outros sistemas de classificação, instrumentos/escalas e elementos inovadores poderão ser desenvolvidos e testados para fortalecer o cuidado no contexto de risco cardiovascular.

## CONSIDERAÇÕES FINAIS

Desenvolveu-se uma TMA que tem como finalidade descrever o cuidado no contexto de risco cardiovascular, a relação dos fatores condicionantes e associados e também prescrever ações para promover a saúde e a redução do risco para reduzir a morbimortalidade por doenças crônicas. A aplicação das estratégias de análise de conceitos e indução teórica a partir do subconjunto terminológico da CIPE^®^ demonstrou a potencialidade de construção de conhecimento próprio oriundo do processo de enfermagem, gerando proposições, pressupostos e modelagem, os quais possibilitarão testagem teórica. Destacam-se a originalidade, o ineditismo e a inovação para o pragmatismo no cuidado para reduzir a morbimortalidade por doenças cardiovasculares a médio e longo prazo da população mundial.
